# The Effect of Synthetic Polymer Foams on Cellulosic Material Degradation

**DOI:** 10.3390/ma16031210

**Published:** 2023-01-31

**Authors:** Vítězslav Knotek, Michal Ďurovič, Irena Kučerová

**Affiliations:** Department of Chemical Technology of Monument Conservation, University of Chemistry and Technology Prague, Technická 5, 166 28 Prague, Czech Republic

**Keywords:** polyethylene foam, cross-infection, SPME-GC/MS, cellulose

## Abstract

Polymeric materials are widely used at present as auxiliary materials in the preservation of tangible cultural heritage in museums, galleries, or libraries. The desired properties of such materials include chemical inertness and long lifetime, which are verified by accelerated aging tests. This work has tested three color types of PE foam Plastazote^®^ LD45 (white, grey, and black), which is recommended for use in the field of cultural heritage preservation. The volatile organic compounds released from the foams after artificial aging and their influence on lignocellulosic materials were investigated. The cellulosic materials were artificially aged in close contact with the foams. All foams released organic acids ranging from acetic acid to hexanoic acid. White foam released organic acids to an extent higher by an order of magnitude compared to the grey and black types. A great influence of white foam on the properties of cellulosic materials was observed. There were large differences in the rate of degradation between the types of foams tested, indicating the need to develop a test method in order to determine the suitability for use in the preservation of culture heritage objects.

## 1. Introduction

Polyethylene (PE) is produced as a copolymer of ethylene with α-olefins (1-butene, 1-hexene, and others), thus achieving short branching, which makes it possible to prepare PE of various crystallinity. The more branched PE is, the lower its density and crystallinity and the higher its sensitivity to oxidation. PE is a thermoplastic, and its properties can be adjusted by cross-linking. As a result of the cross-linking process, the degree of crystallinity of PE decreases. Therefore, the properties associated with the degree of crystallinity (e.g., modulus of elasticity and hardness) are lower compared to non-cross-linked polyethylene [1,2]. Polymeric foams represent a two-phase system in which a gas is dispersed in a solid plastic. The production of an expanded PE foam can be summarized as follows: PE must be melted so that it can be mixed with a blowing agent and possibly other additives. The melt pressed into the desired shape of the product is then cross-linked, and after that, the cross-linked product is exposed to a temperature at which the blowing agent is decomposed. Azodicarbonamide is used as the blowing agent; its decomposition temperature of approximately 200 °C can be reduced with admixtures [1,2,3,4,5].

PE cross-linking is performed by radiation or using peroxides and vinylsilanes [2]. Radiation cross-linking is very fast, takes place at room temperature, and no additives are required for cross-linking; its efficiency is very high. The size of the cells may be regulated by the radiation dose, and a foam product with a density gradient can be produced [1,2,4]. Peroxide cross-linking makes use of a radical mechanism, with the source of the radicals being peroxides, which decompose at increased temperatures. The peroxide cross-linking is influenced by the temperature, type, and concentration of the peroxide used, the molecular mass of PE, the distribution of branching, the presence of a coagent, and other factors. Coagents are low-molecular substances with one or two reactive double bonds that ensure more reactive places, thus increasing the efficiency of cross-linking. Their addition reduces the time needed for cross-linking; they improve thermal aging, peroxide efficiency, and flexibility and ensure higher tensile strength and hardness [1,2]. The first step in the process of cross-linking using vinylsilanes is the grafting of silanes to the PE chain in the melt using peroxide as an initiator. The cross-linking of grafted PE is completed by condensation in the presence of moisture and a catalyst (e.g., dibutyltin laurate), which activates and accelerates condensation. Cross-linking usually takes place at increased temperatures (50–80 °C). Radiation and peroxide cross-linking create cross-link bonds between carbon atoms in neighboring PE chains. In the case of cross-linking using silanes, the cross-link bond is formed by a -Si-O-Si- bridge, which is weaker than a cross-linked bond between two carbon atoms. This affects the strength and long-term thermal stability of the resulting foam [1,2]. Cross-linking using vinylsilanes introduces PE heteroatoms into the chain; their presence reduces the resistance of the polymer to hydrolysis.

In addition to additives that are used to cross-link and expand PE, additives that improve the workability and utilization properties are also added to the mixture. Most additives are added in small quantities and are usually not included in the technical sheets by manufacturers. The additive most frequently used is carbon black, which has an effect as a thermal antioxidant and UV absorber. Antioxidants protect PE from high temperatures in the extruder and during the expansion. In addition, carbon black, zinc oxide, or variously substituted o-hydroxybenzophenones are also used as UV absorbers. Pigments or higher molecular organic dyes are used to adjust the foam’s color. The addition of fatty acid esters improves the workability and quality of the foam [1,2,6,7,8,9,10].

Polyethylene foams made of cross-linked closed-cell polyethylene (PE) are commonly used today when storing and exhibiting sensitive materials such as textile materials, parchment, paper, metal objects, etc. [11,12,13,14]. Thus, the “auxiliary” polymeric materials used must be stable for a long time and must not contaminate the objects or damage them with their additives or with volatile organic compounds (VOC) that originate from their aging. We need to keep in mind that these polymeric materials are susceptible to degradation as other polymeric materials. The speed of their degradation depends on their chemical composition and structure, the additives present, the method of processing the polymer, the ratio between the surface and the volume of the polymeric material, mechanical stress during use, and the conditions of deposition or exhibition [9,15,16,17]. The production of polyethylene is the first stage for its possible oxidation. Oxidation starts in the amorphous regions of the polymer and can spread to neighboring regions [18]. VOCs produced by an oxidized polymer can initiate or accelerate the degradation of polymers stored in its vicinity [19,20]. This phenomenon is known as cross-infection and was first experimentally demonstrated in 2006 [21].

This is why Curran et al. [22] discussed the so-called cross-infection of cellulose with various polymeric materials. Among the tested materials, they also included cross-linked PE foam of the Plastazote brand, which they exposed to the temperature of 80 °C for a period of 14 days. Then they determined cross-infection on the basis of a decline in the degree of polymerization of cellulose after its contact with accelerated-aged Plastazote foam. They found only a moderate influence of Plastazote foams on cellulose. Unfortunately, they did not analyze VOCs released by foams.

Plastazote foams have been recommended for the storage and display of heritage objects for nearly two decades [23], although it has been known for a long time that highly degraded polyethylene can release organic acids [24]. Therefore, in another work, Curran et al. [25] attempted to determine the level of acetic acid released from materials used for the deposition and exhibition of collection items. In their study, they also tested cross-linked PE foam of the Plastazote brand, in particular, samples of white, grey, and blue foam and two samples of black foam. They found out that the studied samples of cross-linked Plastazote foam (except for a black foam sample) release acetic acid even at room temperature and set its concentration within the range of 222–346 ppb. This categorizes Plastazote cross-linked PE foams as high-level VOC emitting polymers according to the Getty Conservation Institute definition. Nevertheless, the authors note that the detected acetic acid could have been absorbed by the foam from the deposition environment [25]. This note is legitimate because some collection items (wood, paper documents) can release acetic acid [26].

Although Plastazote foam is widely used in direct contact with cellulose-based collection objects, the foam has not yet been tested by accelerated aging in direct contact with cellulosic material at elevated humidity. At the same time, the effect of Plastazote foam on the degradation of wood-pulp papers has not yet been evaluated. In the case of papers containing lignin, humidity is more of a degradation factor than low levels of acetic acid [27]. Therefore, the aim of this work is to evaluate the influence of Plastazote foam on cellulosic materials in direct contact at elevated humidity and to verify the release of VOCs caused only by the degradation of Plastazote foams.

## 2. Materials and Methods

### 2.1. Tested Materials

Brand new Plastazote^®^ LD45 polyethylene foam sheets were purchased from Conservation By Design Ltd. (CBD, Milton Keynes, UK). Three color types of Plastazote^®^ LD45 boards, white, grey, and black, were tested. The delivered boards had dimensions of 2000 × 1000 × 12 mm. According to the CBD information, Plastazote^®^ is a cross-linked polyethylene foam with a closed cell structure, physically expanded with nitrogen. Plastazote^®^ should not contain any blowing agent residues.

As representatives of lignocellulosic materials, Whatman filter paper (Grade 1; 100% pulp, basis weight: 87 g/m^2^, supplier VWR International, Stříbrná Skalice, Czech Republic) and wood-pulp paper (mixture of wood pulp and unbleached sulphite pulp, smoothed on one side, sized with alum and resin, basis weight: 60 g/m^2^, supplier of paper mill Větřní, Czech Republic) were tested.

### 2.2. Artificial Aging

**Figure 1:**
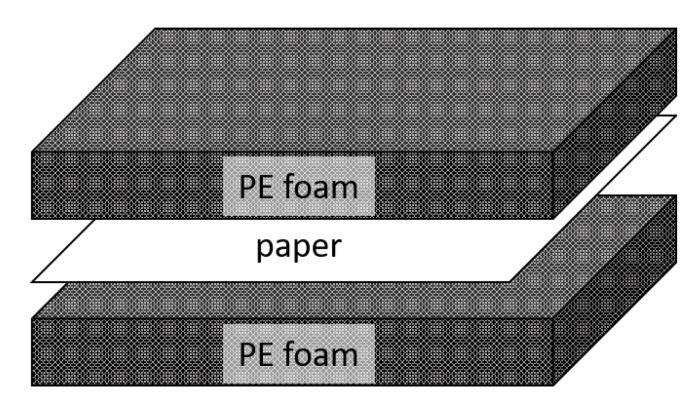
The designation of sandwiches for artificial aging.

To determine the effect of PE foam on lignocellulosic materials, two A4-sized Plastazote LD45 boards were prepared, between which a sheet of Whatman paper and a sheet of wood-pulp paper of the same size was inserted. The resulting sandwiches (Figure 1) were artificially aged in a Memmert HCP 108 climate chamber at 80 °C and 65% relative humidity for 28 days. At the same time, individual sheets of Whatman paper and wood pulp paper were aged in a separate climate chamber under the same conditions as the sandwiches.

To detect cross-infection, PE foams were tested according to the procedure described in the literature [22], where a sample of PE foam and Whatman paper was placed in a Schott glass vial that was hermetically sealed. Schott glass vials with samples were placed in an oven at 80 °C for 14 days. In the same way, the PE foam samples were aged separately without the presence of Whatman paper. These samples were used to identify VOCs released from PE foams (see Section 2.3.3). To avoid the influence of possible gaseous impurities adsorbed by the PE foams during the storage period, each PE foam board was heated for 1 h at 60 °C prior to testing.

### 2.3. Properties of PE Foams

#### 2.3.1. Composition

Differences in the composition of different colored foams were investigated using X-ray fluorescence with a wave-dispersive detector (WD-XRF, PANalytical Axios, PANalytical B.V., Almelo, The Netherlands), X-ray diffraction (Cu anode, 30 mA, 40 kV, 5–90°, PANalytical X’Pert PRO, PANalytical B.V., Almelo, The Netherlands) and FTIR spectroscopy using the attenuated total reflection technique (ATR-FTIR, Nicolet iZ10, Thermo Scientific, Walthamm, MA, USA, diamond crystal, resolution 4 cm^−1^, number of scans 64). Changes in the chemical composition of PE foams after artificial aging were investigated with ATR-FTIR spectroscopy.

#### 2.3.2. Mechanical Properties

The degree of elasticity of the PE foams after artificial aging was assessed by determining the compression set at ambient temperature in a laboratory. Testing was carried out according to the DIN ISO 815:1991 standard–Determination of compression set at ambient temperature. The average value and standard deviation of ten values for each foam were evaluated and rounded to the nearest percentage unit. The compression set *C* was calculated according to Equation (1):

(1) $$C=\frac{h_{0}-h_{1}}{h_{0}-h_{s}}\times 100,$$

where *h*_0_ is the initial thickness of the test piece, *h*_1_ is the thickness of the test piece after recovery, and *h_s_* is the height of the spacer. The height of the spacer was the same for all the tested PE foams.

#### 2.3.3. VOC Emissions Analysis

Samples of foams weighing 200 ± 5 mg were removed from the Schott glass vials after aging and immediately transferred to 60 mL brown headspace glass vials and sealed with a screw cap ND24 (EPA) with a silicone/PTFE liner. The vials were left in the dark at room temperature for 14 days prior to analysis. The extraction of VOCs was carried out using a DVB/CAR/PDMS SPME fiber (50/30 μm, 57298-U, Supelco, St. Louis, MO, USA). The absorption times were 60 min at 23 °C. A gas chromatograph (TRACE GC ULTRA, Thermo Scientific, Walthamm, MA, USA) coupled to an ISQ mass spectrometer (Thermo Scientific) was used to recover and analyze the substances from the fiber. The injector temperature was 250 °C, and the injector was used in the split mode of 1:10. The carrier gas was helium 6.0 with a constant flow rate of 1.5 mL/min. A DB-5MS UI column (60 m × 0.32 mm, film 1.0 μm) was used for the separation of the VOCs using a temperature program as follows: starting temperature of 40 °C (hold for 5 min), a constant temperature increase at a rate of 15 °C/min until the final temperature of 290 °C (hold for 5 min).

Mass spectra were collected in the electron ionization (EI) mode at 70 eV in the range of 29–450 Da. The scan time was 0.25 s. The VOCs peaks were identified using the NIST 20 database of MS spectra.

### 2.4. Properties of Lignocellulosic Materials

#### 2.4.1. Colorimetry

The color of the sandwich papers was measured with a CM-700d spectrophotometer (Konica Minolta, Tokyo, Japan) in the CIE L*a*b* color space [28]. The spectrophotometer was controlled using the SpectraMagic NX program. The total color difference ΔE* was calculated using Equation (2), where $L_{0}^{*}$, $a_{0}^{*}$, $b_{0}^{*}$ are the colour parameters before disinfection and before artificial aging and $L_{1}^{*}$, $a_{1}^{*}$, $b_{1}^{*}$ are the colour parameters measured after artificial aging.

(2) $$\Delta E^{*}=\sqrt{{\left(L_{1}^{*}-L_{0}^{*}\right)}^{2}+{\left(a_{1}^{*}-a_{0}^{*}\right)}^{2}+{\left(b_{1}^{*}-b_{0}^{*}\right)}^{2}}$$

Five measurements were evaluated on each side of each paper. The average value and standard deviation of ten values were evaluated for each paper.

#### 2.4.2. UV/Vis Spectroscopy

Reflection spectroscopy was used in the UV and Vis regions to obtain a detailed evaluation of the changes in the chromophore systems of the samples after irradiation and artificial aging. Measurements were performed on a Cary 60 (Agilent, Santa Clara, CA, USA) UV/Vis spectrometer with an integration sphere for measuring the diffusion reflectance (Barrellino). The reflection spectra were measured at wavelengths of 200–800 nm. The spectra of each sample were measured ten times. The K/S curves and decoloration numbers DC_λ_ were calculated from the measured values according to Equations (3) and (4).

The ratio factor K/S expresses the ratio of the light absorbance coefficient K, and the light scattering coefficient S. The relationship between the reflectivity R_∞_ and the coefficient of light absorbance and scattering is defined by the Kubelka-Munk Equation (3).

(3) $$\frac{K}{S}=\frac{{\left(1-\frac{R_{\infty }}{100}\right)}^{2}}{2\;\cdot \frac{R_{\infty }}{100}}$$

The decoloration number DC_λ_ expresses the change in the hue of the sample during degradation. When it is lighter, the value of DC_λ_ is positive; when it is darker, it is negative. The decoloration curve can be used to assess the formation of chromophore systems after irradiation and/or artificial aging.

(4) DCλ=KSOλ−KSAλ ,

where the ratio factor KSOλ corresponds to the original sample and KSAλ to the sample after the relevant decoloration change.

#### 2.4.3. pH Value of an Aqueous Extract

The pH of a cold aqueous extract was determined using an inoLab 7310 pH meter and a Sentix 41 glass electrode (WTW GmbH, Weilheim, Germany) according to standard ISO 6588. The resultant value is the average of two determinations.

#### 2.4.4. Mechanical Properties

The breaking length was measured on an Alvetron TH1 instrument (Lorentzen & Wettre, Stockholm, Sweden). The zero-span breaking length was measured on a LabTest 5.030 instrument (LaborTech, Opava, Czech Republic). The listed results are the average of 10 measurements. The results were processed statistically, and the error is expressed as the corrected sample standard deviation.

#### 2.4.5. Average Degree of Polymerization

The measurement of the limiting viscosity number [η] was used to monitor any cleavage of macromolecules. For cellulosic samples, it is feasible to calculate the average degree of polymerization (DP) from [η]. The limiting viscosity number was determined using a capillary Ubbelohde viscometer.

A bis(ethylenediamine)copper(II) hydroxide solution (CED) was used to dissolve the paper samples, and the determination was carried out according to ISO 5351/1. Two determinations were performed for each sample, and the average degree of polymerization was calculated from Equation (5).

DP^0.85^ = 1.1∙[η] (5)

## 3. Results

### 3.1. PE Foams

#### 3.1.1. Composition

**Figure 2:**
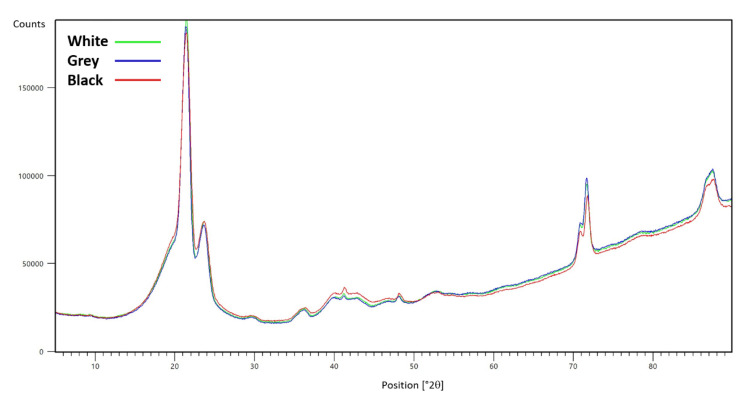
Comparison of XRD curves for white, grey, and black PE foam.

X-ray diffraction analysis was performed to discover prospective differences in the content of crystalline substances (such as pigments). A comparison of X-ray diffractograms for the tested PE foams is presented in Figure 2.

X-ray fluorescence analysis was carried out to discover the content of elements heavier than fluorine in the PE foams. The result of the analyses normalized to 100% for the individual foams is presented in Table 1. The contents of the individual elements are low and almost identical for white and black foam. In the case of grey foam, some elements were not detected at all, or their content was lower.

**Figure 3:**
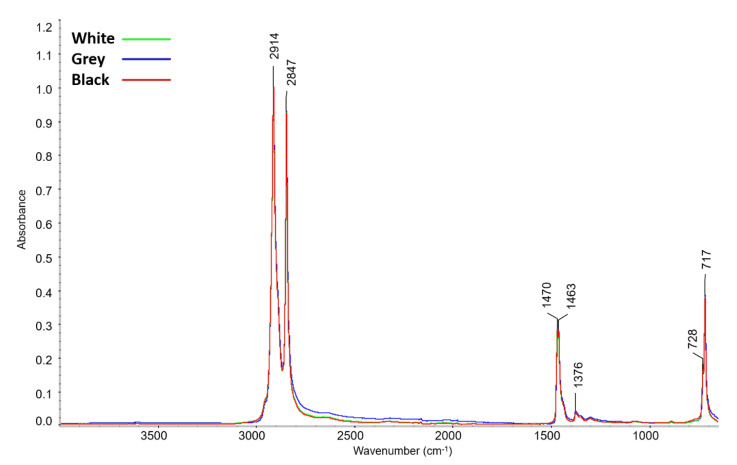
ATR-FTIR spectra of the tested PE foams before artificial aging.

Before artificial aging was performed, the composition of the foams was examined by infrared spectroscopy. The results showed identical spectra for all examined foams (see Figure 3). No signs of oxidation of the foams were observed.

**Figure 4:**
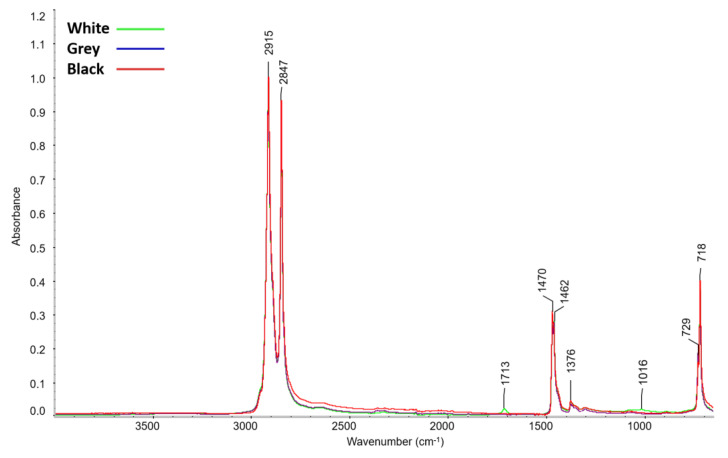
ATR-FTIR spectra of the tested PE foams after artificial ageing.

**Figure 5:**
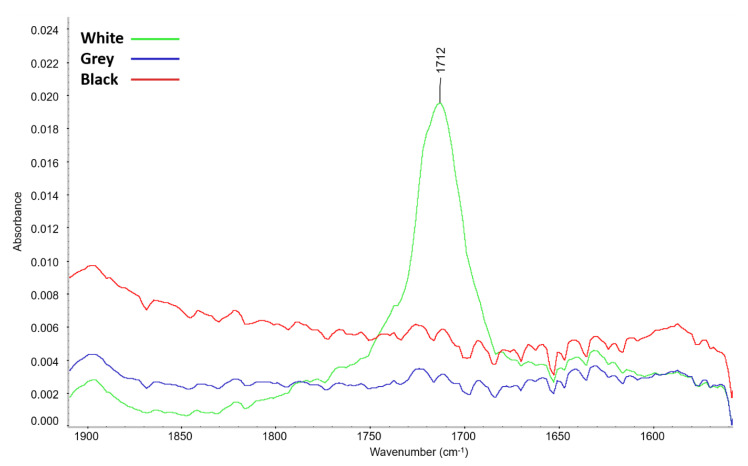
Details of the 1900–1550 cm^−1^ range of ATR-FTIR spectra of the tested PE foams after artificial aging.

**Figure 6:**
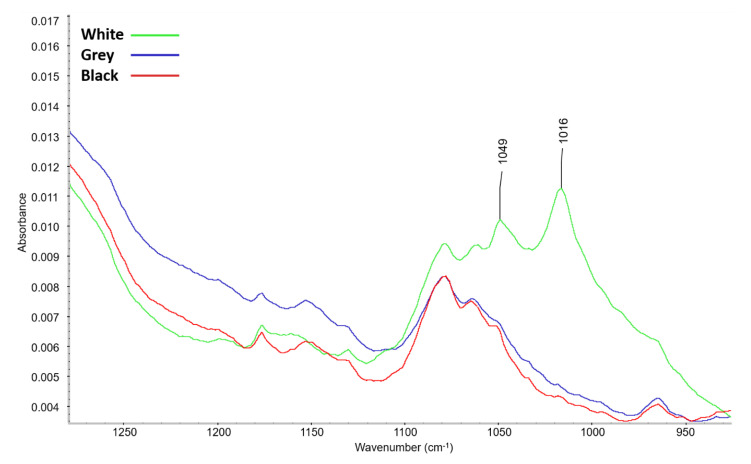
Details of the 1200–900 cm^−1^ range of ATR-FTIR spectra of the tested PE foams after artificial aging.

Figure 4 shows a comparison of the infrared spectra of the PE foams after artificial aging. The formation of new peaks was only recorded for white foam. In view of the low intensity of these new peaks, details of the spectra are shown in Figure 5 and Figure 6. The maxima of the new peaks for white foam corresponded to 1712, 1049, and 1016 cm^−1^. The new peaks can be assigned to oxygen-containing groups, namely 1712 cm^−1^ for the carbonyl group (C=O) and 1049 cm^−1^ together with 1016 cm^−1^ for the ether structure (C-O-C).

#### 3.1.2. Mechanical Properties

Table 2 presents the compression set results for the foams after artificial moist heat aging. The compression set expresses the percentage of loss of thickness of the specimen after the removal of pressure loading in relation to the original dimensions. A higher figure in the results indicates a lower ability to return to the original size after compression.

#### 3.1.3. Volatile Organic Compounds (VOC)

**Figure 7:**
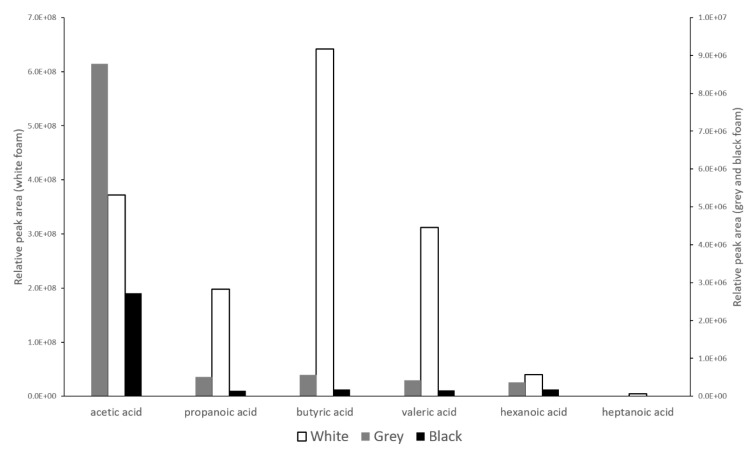
Relative ratios of the peak areas for organic acids detected by GC/MS.

VOCs released from the foam samples after artificial aging in closed vials were determined using GC/MS. An overview of the compounds detected is presented in Table 3. It shows that for black foam, hydrocarbons such as decane, undecane, and dodecane were detected, above all, after aging. Acetic acid was present in a small quantity. The results for grey foam were similar. A great difference in the composition of VOCs was observed for the white foam sample, where a higher content of acetic, propanoic, butyric, and valeric acid was discovered. No hydrocarbons were detected for the white sample, only the respective ketones or aldehydes. A very similar composition of VOCs was detected in strongly degraded PE films caused by photo-oxidation and thermo-oxidation processes [24]. Figure 7 shows a graphic comparison of the areas under the acids’ peaks for the individual samples.

Organic acids ranging from acetic acid to hexanoic acid were detected for all foams. Heptanoic acid was detected for white PE foam. Figure 7 shows that acetic acid was the most represented acid for grey and black foam. White foam released the greatest quantity of butyric and acetic acids. The quantity of acetic acid released by the white foam was approximately 100 times higher compared to the black foam. The emissions of other acids by white foam were also higher by an order of magnitude compared to those of grey and black foam.

### 3.2. Lignocellulosic Materials

#### 3.2.1. Colorimetry

**Figure 8:**
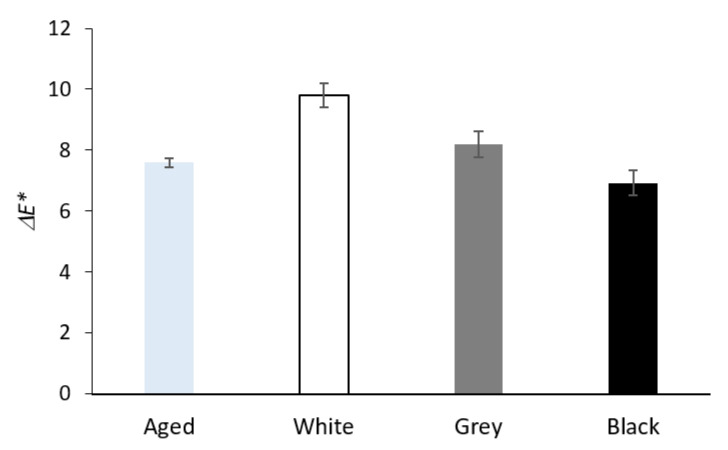
The total color difference of Whatman Grade 1 paper after artificial aging without contact with PE foam (Aged) and in contact with the tested PE foams.

Figure 8 shows the total color difference of Whatman paper after the artificial aging of a sheet separately and placed between PE foam boards (sandwiched). The greatest change in color difference was recorded for paper in contact with white foam; the biggest change had the form of higher values of the b* coordinate, which means yellowing. Compared to the aging of a separate sheet of Whatman paper, the sandwiched papers showed higher Δb* values for all three types of PE foams. Paper in contact with grey and black foam showed an overall change in the total color difference comparable to paper that aged separately. A darkening (a negative ΔL* value) took place for the separately aged Whatman paper sheet. The darkening was lower for all sandwiched papers. The lowest darkening was recorded for paper sandwiched in black foam.

**Figure 9:**
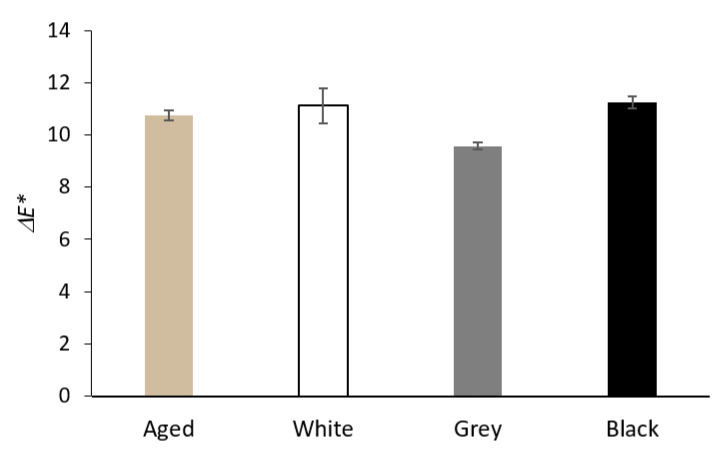
The total color difference of wood-pulp paper after artificial aging without contact with PE foam (Aged) and in contact with the tested PE foams.

No significant changes in the total color difference were recorded for wood-pulp paper after artificial aging as a result of contact with PE foams, as shown in Figure 9. Compared to separately aged wood-pulp paper, the sandwiched paper showed slight yellowing for all tested PE foams, most of all for white foam.

#### 3.2.2. UV/Vis Spectroscopy

**Figure 10:**
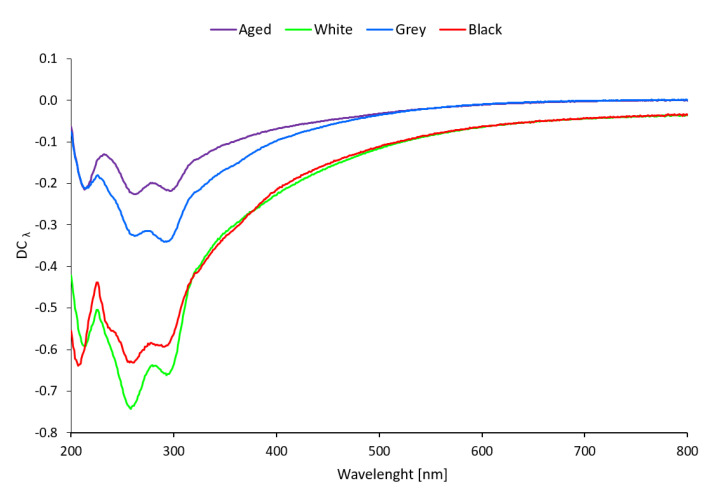
Decoloration curves for Whatman Grade 1 paper aged separately (Aged) and sandwiched in PE foam.

Figure 10 shows the decoloration curves for Whatman paper aged separately and sandwiched between PE foam boards. Three distinct minima at the wavelengths of 217, 260, and 296 nm can be seen in all the curves, indicating the formation of chromophores or chromogens because their absorption also reaches into the visible part of the spectrum. Figure 10 makes it evident that the formation of cellulose chromogens is most supported by white PE foam, followed by black and, to the least extent, grey PE foam.

#### 3.2.3. pH Value of an Aqueous Extract

The pH values of a cold extract of the tested papers are summarized in Table 4. For the Whatman paper, the aging was followed by an approximately tenfold increase in the concentration of hydrogen ions to a pH of 5.3. When aged in sandwiches, the pH of the extract for grey and black foam was approximately equal to that of paper without contact with PE foam. For the Whatman paper, the sample sandwiched in white foam showed the lowest pH value of the extract.

For wood-pulp paper, the aging led to a slight increase in pH for the sample without contact with PE foam. The samples of wood-pulp paper in contact with PE foams showed the same pH as a cold extract. The pH value decreased compared to the sample aged separately.

#### 3.2.4. Mechanical Properties

**Figure 11:**
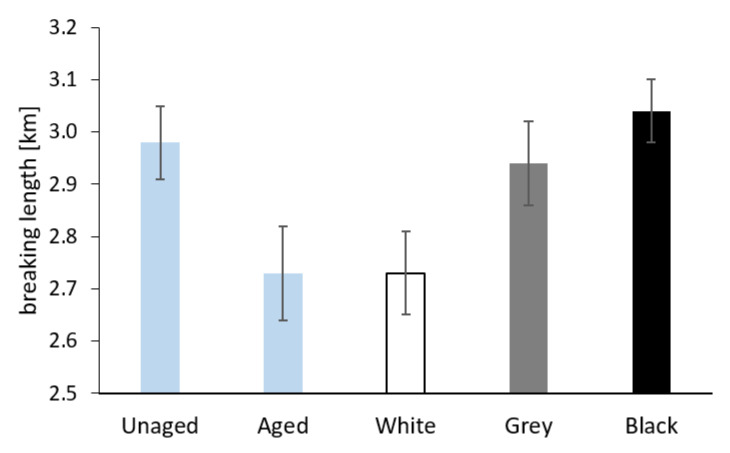
The breaking length of Whatman Grade 1 paper before aging (Unaged), after artificial aging without contact with PE foam (Aged), and in contact with the tested PE foams.

**Figure 12:**
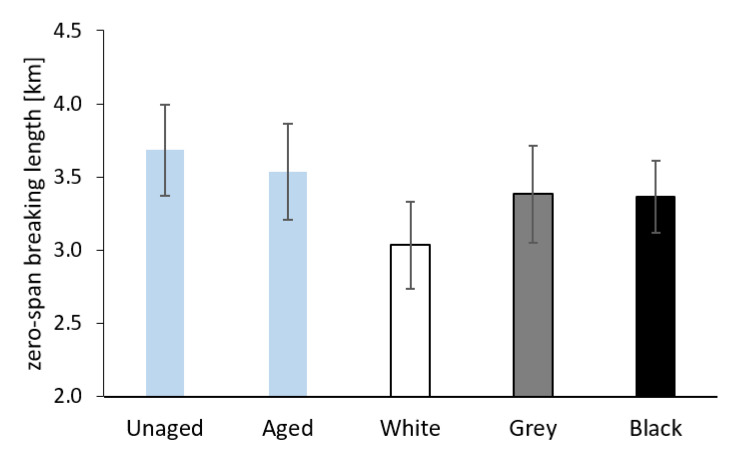
The zero-span breaking length of Whatman Grade 1 paper before aging (Unaged), after artificial aging without contact with PE foam (Aged), and in contact with the tested PE foams.

The results of the measurement of the breaking length and the zero-span breaking length for the Whatman paper before and after aging, both separately and sandwiched, are shown in Figure 11 and Figure 12. The values of both the breaking length and the zero-span breaking length decreased after separate aging of the Whatman paper. In the case of sandwiched paper, the samples aged between white PE foam showed the lowest mechanical properties. The samples of paper from sandwiches with grey and black foam had breaking lengths comparable to paper before aging. As for zero-span breaking length, the average values for paper from the grey and black sandwich were slightly lower compared to the sample of paper aged separately.

**Figure 13:**
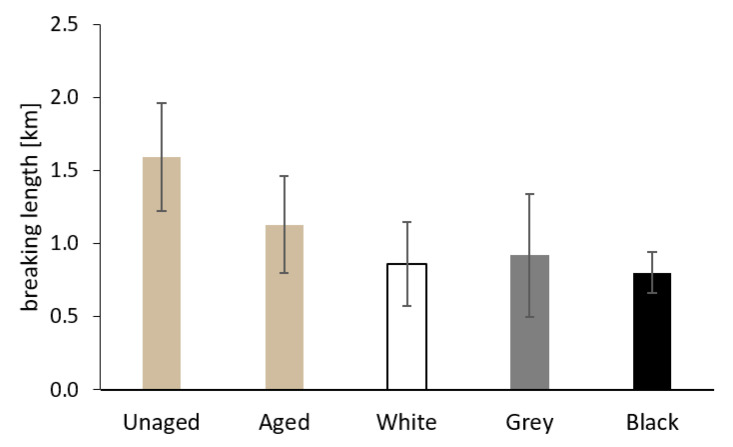
The breaking length of wood-pulp paper before aging (Unaged), after artificial aging without contact with PE foam (Aged), and in contact with the tested PE foams.

**Figure 14:**
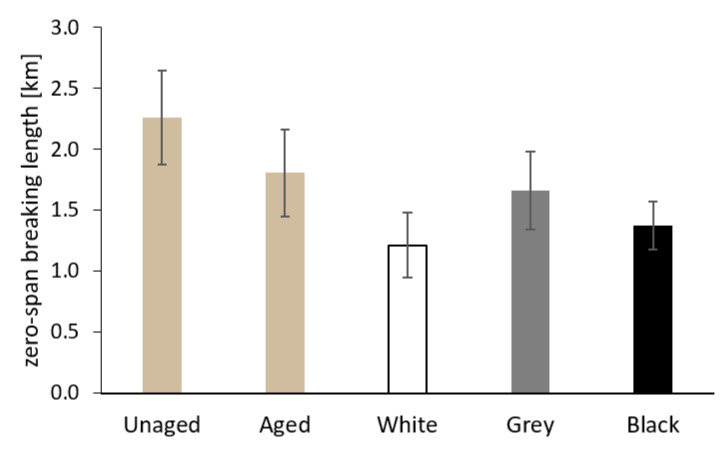
The zero-span breaking length of wood-pulp paper before aging (Unaged), after artificial aging without contact with PE foam (Aged), and in contact with the tested PE foams.

The results of the measurement of the breaking length and zero-span breaking length for wood-pulp paper before and after aging, both separately and sandwiched, are shown in Figure 13 and Figure 14. The breaking length values for wood-pulp paper aged in sandwiches are almost the same for all three PE foams and approximately a quarter lower compared to the sample aged without contact with PE foam.

The lowest value of zero-span breaking length was measured for the sample from the white foam sandwich. The value of the sample from the grey foam sandwich was the highest of the sandwiched samples, approaching the value of the sample aged separately.

#### 3.2.5. Average Degree of Polymerization

**Figure 15:**
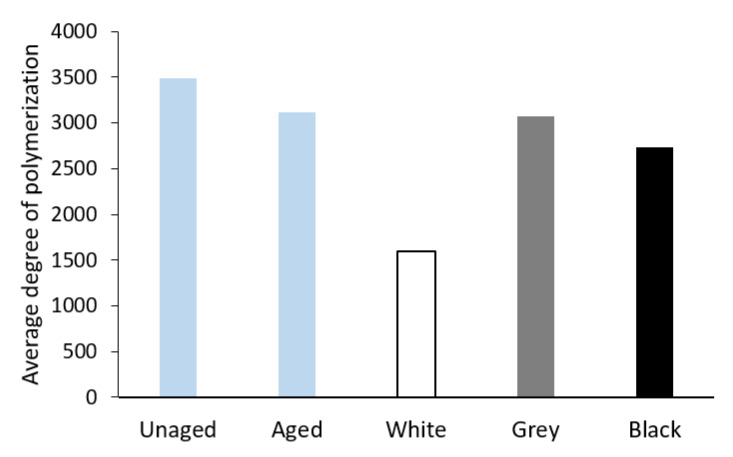
The average degree of polymerization of Whatman Grade 1 paper before aging (Unaged), after artificial aging without contact with PE foam (Aged), and in contact with the tested PE foams.

Due to the poor solubility of wood-pulp paper, the average degree of polymerization (DP) was only measured for the Whatman paper samples. A slight decrease in DP for separately aged paper can be seen in Figure 15. The highest decline in DP, approximately one-half, was recorded for the sample from the white foam sandwich. On the contrary, the DP of the grey foam sandwich sample was almost the same as for the sample without contact with PE foam. Paper aged in the black foam sandwich showed the DP lower by approximately 300.

## 4. Discussion

No significant differences were discovered when examining the composition of the PE foams by XRF, XRD, and ATR-FTIR. No substances of crystalline character were detected in any PE foam. According to a statement from Zotefoams plc, the manufacturer of Plastazote^®^ foam, the tested foams only differ in the carbon black content, which is present with the highest quantity in black foam and not at all in white foam. The results of the analyses indirectly confirm this information since the used XRF and FTIR methods cannot detect carbon. In the case of XRD, it is difficult to detect small amounts of amorphous or nanocrystalline carbon.

The content of a small amount of sulphur detected by XRF analysis for white and black foam may indicate the presence of thioesters, which are used as stabilizers suppressing thermo-oxidation. The content of nickel and iron may also be related to the use of a specific stabilizer [24,29]. Generally, a wider range of efficient antioxidants can be used for black plastics, as some of them change color, an effect that is not observable in black objects [29]. In addition to the coloring effect, carbon black also has a stabilization effect against photo-oxidation. Silicon detected in the elemental composition may be related to the cross-linking means used on the basis of vinylsilanes. However, XRF did not detect any tin, which is used in the form of organic-tin compounds as a catalyst for the condensation of silanes.

In contrast to the small differences in the composition, considerable differences in the VOCs released by the aged foams were detected. The aging was accompanied by reactions to oxidation or even the formation of organic acids. PE foams are known to be able to release low concentrations of hydrocarbons of various lengths [30]. In the presence of oxygen, the hydrocarbons may oxidize to the respective aldehydes or ketones. The last degree of oxidation is carboxylic acid. Of the tested foams, the white foam was the least resistant to oxidation since only aldehydes, ketones, and acids were detected among the released compounds. Such an extent of hydrocarbon oxidation products was not released for grey and black foam. Their resistance to oxidation is higher compared to white foam. This statement is confirmed by a semi-quantitative comparison of the released carboxylic acids (see Figure 7). Quantities of acetic, propanoic, butyric, or valeric acid that were greater by an order of magnitude were released for white foam. It can be assumed that the detected acetic acid in [25] came mainly from degraded Plastazote foam. Acetic acid is considered a very dangerous pollutant in the preservation of a wide spectrum of materials such as paper documents, leather, limestone, copper alloys, zinc, or hydrolyzable synthetic polymers [22,27,31,32,33].

Volatile compounds based on silanes were detected for all foams. The presence of silanes may be related to the cross-linking process [2] because, according to the manufacturer’s information, Plastazote^®^ PE foam is cross-linked.

The presence of the carboxylic group for aged white foam is also confirmed by the FTIR spectra, where a new peak with a maximum of 1712 cm^−1^ was detected, corresponding to the ν(C=O) vibration of carbonyl present in a carboxylic acid [34]. Peaks with 1049 and 1016 cm^−1^ maxima corresponding to the ν(C-O-C) vibration were also identified. The ATR-FTIR spectra of grey and black foam were unchanged after aging.

The differences among the foams in the level of oxidation were reflected in their mechanical properties, with white foam showing a slightly higher compression set than the grey and black foam (see Table 2). A higher compression set signifies a loss of elastic capabilities, which is probably related to the shortening of polymeric chains. The greater amount of released volatile compounds for white foam indicates a higher quantity of cleaved C-C bonds. As a result of the formation of a new constitution of the polymer with shorter chains and a smaller quantity of short branching, it no longer achieves dimensional constancy after pressure loading. These results outline a way to test the condition of Plastazote^®^ PE foam easily.

Whatman paper is 100% pulp, a material susceptible to oxidation and hydrolysis at increased temperature and humidity, manifested by a decrease in the degree of substitution and the release of furan derivatives and organic acids [31,35,36,37]. A considerable change of color, caused, above all, by yellowing, was recorded after separate aging of the Whatman paper. The change of color was the greatest in contact with Whatman paper with white foam due to more considerable yellowing (see Figure 8). Yellowing of polymers is associated with degradation processes, especially oxidation when oxidized structures come into existence on the surface [38,39]. Contact with grey and black foam led to similar color changes as in the case of separately aged Whatman paper. The presence of oxidation products on the surface of the Whatman paper after artificial aging was confirmed by the results of UV/Vis analysis. The resulting minima in UV/Vis curves (see Figure 10) signal the formation of new chromophores by the oxidation of hydroxyl groups of β-D-glucopyranose. Ketone functional groups on C2 or C3 carbon atoms of β-D-glucopyranose absorb at about 260 nm, while ketone groups on C2 and C3 β-D-glucopyranose (diketones) or aldehyde groups of the furan cycle absorb at higher wavelengths (approx. 300 nm). C=C-C=O structures (furfuraldehydes) are the product of the elimination of a hydroxyl group in β–position to a carbonyl group, so-called β-elimination [40,41,42].

Artificial aging led to a decrease in pH in the Whatman paper, signaling ongoing degradation processes connected with the formation of acidic products. The lowest pH of a cold extract in the sample from the white foam sandwich may be caused by the absorption of acids released from the white foam by the paper or by a greater extent of paper degradation caused by the effect of a higher concentration of acidic substances [37]. In the presence of moisture, an acidic environment supports acid hydrolysis of cellulose, which manifests itself above all by a shortening of the polymeric chain [31,32,35,43].

The degradation processes of cellulose should also manifest themselves in the results of the mechanical properties tests. The value of the breaking length of the paper is related both to the average degree of polymerization and to the physical interactions between macromolecules. For the Whatman paper, the aging in grey and black foam sandwiches had a positive influence on the breaking length value compared to the results for paper aged separately and paper from the white sandwich (see Figure 11). It seems that the restriction of airflow around the paper caused by its surrounding stable material (grey and black sandwich) has a positive effect on its breaking length.

The value of the zero-span breaking length of the paper is, in general, dependent on the average degree of polymerization of cellulose [44] and, as the case may be, of other contained macromolecules. Indeed, the average values of the zero-span breaking length for the Whatman paper (see Figure 12) correlate very well with the average degree of polymerization value measured viscometrically (see Figure 15). The organic acids released from the white foam caused a considerable decrease in the average degree of polymerization as a consequence of acid hydrolysis. Tétreault et al. [44] say that the average degree of polymerization (DP) depends on the value of the b* component in the CIE L*a*b* color space and the pH value of a cold extract. The correlation between DP, the b* value, and pH is confirmed by the results of the Whatman paper in the present work.

Wood-pulp paper showed a very high color change after aging, and similar results were observed after aging in foam sandwiches. Wood-pulp paper is a mixture of cellulose, lignin, and other substances present in the wood. The color change is the sum of chemical changes in the individual components. An analysis of the contribution of the individual components of the L*a*b* color space to the total color difference implied a slight yellowing of the samples enclosed in sandwiches. The samples of wood-pulp paper from sandwiches showed lower values of pH (Table 4) and breaking length (Figure 13). The reason may be the release of acidic products from wood-pulp paper [45] during aging and the possibility of their concentrating and subsequent degradation effect on the paper enclosed between the PE foam boards. The negative effect of the acidic products of the white foam manifested itself in the results of the zero-span breaking length (see Figure 14). In connection with the results for the Whatman paper, the greatest decline in DP presumably also occurred after aging wood-pulp paper in a white sandwich.

The influence of selected types of VOCs (formic acid, acetic acid, formaldehyde, acetaldehyde, hexanal, furfural, vanillin) on cellulosic materials was intensively studied [27,37]. However, degraded PE foam may release various VOCs (e.g., propanoic or butyric acid) whose effect on cellulose has not yet been investigated. Another theoretical factor in the degradation of cellulosic materials in contact with PE foam may be the content of trace amounts of heavy metals (see Table 1), which are responsible for accelerating the degradation of cellulose [46].

## 5. Conclusions

The testing of Plastazote^®^ LD 45 PE foam, recommended for use in the preservation of cultural heritage, has revealed rather large differences in resistance to oxidation between the individual color types. After aging, the white PE foam released a range of carboxylic acids, from acetic acid to hexanoic acid. The increased concentration of these acids had a negative effect on the properties of lignocellulosic materials. Carboxylic acids were also detected for the grey and black PE foam. In light of lower manifestations of damage to lignocellulosic materials, the concentration of released acids was lower for grey and black foam. Grey Plastazote^®^ LD45 foam influenced the physical-chemical properties of pure cellulose paper (Whatman Grade 1) and paper with a high content of wood pulp the least. Among the tested foams, the grey type of Plastazote ^®^ LD45 can be recommended for use in conservation practice. From the perspective of practical use, it is advisable to periodically replace the polyethylene foam with a new one in order to minimize the risk of the release of oxidation products in higher concentrations.

This study has shown great differences in the long-term resistance of one type of material recommended for historic preservation. Despite the identical physical properties, differences in stabilization or processing may exist between the individual types of the material. A cross-infection test with Whatman Grade 1 paper can be recommended to determine the suitability of the use of auxiliary materials for contact with sensitive collection items.

## Figures and Tables

**Table 1:** Results of XRF analyses for PE foams.

PE Foam	Element Content (wt.%)											
	Na	Mg	Al	Si	S	Cl	K	Ca	Cr	Fe	Ni	Zn
White	0.043 (0.006) ^1^	0.123 (0.01)	0.010 (0.003)	0.093 (0.009)	0.003 (0.002)	0.020 (0.004)	0.005 (0.002)	0.003 (0.002)	0.003 (0.002)	0.009 (0.003)	0.001 (0.0007)	
Grey	0.003 (0.002)		0.002 (0.001)	0.003 (0.002)		0.007 (0.002)			0.001 (0.0008)	0.002 (0.001)	0.001 (0.0007)	
Black	0.040 (0.006)	0.006 (0.002)	0.021 (0.004)	0.034 (0.005)	0.010 (0.003)	0.026 (0.005)	0.003 (0.002)	0.011 (0.003)	0.003 (0.002)	0.007 (0.003)	0.001 (0.0007)	0.001 (0.0008)

^1^ Numbers in brackets mean Absolute error of the measurement.

**Table 2:** Compression set of PE foams.

PE Foam	Compression Set (%)
White	31 ± 1
Grey	27 ± 1
Black	28 ± 1

**Table 3:** Retention time (RT) and corresponding compounds detected for PE foams after artificial aging.

White Foam		Grey Foam		Black Foam	
RT (Min)	Compound	RT (Min)	Compound	RT (Min)	Compound
4.00	acetaldehyde				
4.72	ethanol	4.72	ethanol	4.73	ethanol
5.41	acetone	5.42	acetone	5.42	acetone
5.49	propionaldehyde				
5.64	formic acid				
6.17	terc.-butanol				
6.91	1-propanol				
7.12	trimethylsilanol	7.13	trimethylsilanol	7.13	trimethylsilanol
7.67	acetic acid	7.34	acetic acid	7.29	acetic acid
7.86	2-butanone				
		9.81	dimethylsilanediol	9.81	dimethylsilanediol
9.49	1-butanol				
10.01	propanoic acid	10.01	propanoic acid	10.01	propanoic acid
10.24	3-pentanone				
10.30	pentanal				
11.42	3-methyl-2-pentanone				
11.63	1-pentanol				
				11.67	methylheptane
11.79	butyric acid	11.79	butyric acid	11.79	butyric acid
11.98	3-hexanone				
12.06	2-hexanone				
12.32	hexamethylcyclotrisiloxane	12.32	hexamethylcyclotrisiloxane	12.32	hexamethylcyclotrisiloxane
				12.63	dimethylheptane
				12.97	dimethylheptene
13.30	valeric acid	13.30	valeric acid	13.30	valeric acid
13.64	3-heptanone				
13.69	2-heptanone				
14.63	hexanoic acid	14.64	hexanoic acid	14.64	hexanoic acid
				14.73	iso-C10H22
14.70	gama-pentalactone				
14.88	4-octanone				
15.04	3-octanone				
15.09	2-octanone				
15.50	iso-C11H24	15.50	iso-C11H24	15.50	iso-C11H24
15.85	heptanoic acid				
16.14	5-nonanone				
16.35	2-nonanone				
		16.44	n-undecane	16.43	n-undecane
16.50	decamethylcyclopentasiloxane	16.50	decamethylcyclopentasiloxane	16.50	decamethylcyclopentasiloxane
17.28	5-decanone				
17.50	2-decanone				
		17.56	n-dodecane	17.55	n-dodecane
				18.59	n-tridecane

**Table 4:** Cold extract pH value for tested types of paper.

	pH Value	
Treatment	Whatman	Wood-Pulp Paper
Unaged	6.2	4.2
Aged	5.3	4.5
White	4.7	4.0
Grey	5.5	4.0
Black	5.5	4.1

## Data Availability

Not applicable.
